# Tirbanibulin 1% Ointment Significantly Reduces the Actinic Keratosis Area and Severity Index in Patients with Actinic Keratosis: Results from a Real-World Study

**DOI:** 10.3390/jcm12144837

**Published:** 2023-07-22

**Authors:** Michael Constantin Kirchberger, Michael Gfesser, Michael Erdmann, Stefan Schliep, Carola Berking, Markus Vincent Heppt

**Affiliations:** 1 Hautarztzentrum Ingolstadt, Schlüterstr. 3a, 85057 Ingolstadt, Germany; 2 Department of Dermatology, Uniklinikum Erlangen, Friedrich-Alexander-University Erlangen-Nürnberg (FAU), Ulmenweg 18, 91054 Erlangen, Germany; 3 Comprehensive Cancer Center Erlangen-European Metropolitan Area of Nuremberg (CCC ER-EMN), 91054 Erlangen, Germany

**Keywords:** actinic keratosis, Klisyri, AKASI score

## Abstract

Background: Actinic keratosis (AK) is a cutaneous lesion resulting from the proliferation of atypical epidermal keratinocytes caused by long-term exposure to ultraviolet radiation. AK may progress to cutaneous squamous cell carcinoma (cSCC) and therefore is often treated with topical agents such as 5-fluorouracil, diclofenac, imiquimod, and photodynamic therapy. Tirbanibulin has been approved based on two phase III trials in the USA. However, real-world evidence for tirbanibulin is absent. Methods: This was a single-centre study of adult patients with clinically typical, visible AK on the face or scalp treated with tirbanibulin 1% ointment. Treatment was administered as per label once daily for 5 consecutive days on the same lesions or field. Treatment outcomes were assessed 4 weeks after treatment, with additional optional assessments conducted at later time points. Efficacy was measured using the actinic keratosis area and severity index (AKASI) and digital dermoscopy. Results: A total of 33 patients were treated of whom 30 were analysed. The median AKASI score was 5.6 (1.4–11) pre-treatment and 1.2 (0–7.4) post-treatment (*p* < 0.0001). Complete clearance as defined by AKASI scores less than 1 was achieved in 47% (*n* = 14) and 57% (*n* = 13) at the first and second follow-up, respectively. All local reactions resolved spontaneously and without sequelae. The most common local reactions were erythema (80%, *n* = 26) and flaking or scaling (43%, *n* = 13). Conclusions: Tirbanibulin 1% ointment significantly and rapidly reduced the AKASI score in a real-world setting. The complete clearance rates were in line with those observed in the two pivotal trials.

## 1. Introduction

Actinic keratosis (AK) is a cutaneous lesion resulting from the proliferation of atypical epidermal keratinocytes. AK lesions typically occur in light-skinned individuals as a result of long-term exposure to ultraviolet radiation [1]. AK can appear as solitary or multiple lesions and present as diffuse red and scaling plaques with a rough surface in highly sun-exposed areas, often with adjacent skin showing signs of chronic solar damage [2]. Distinct clinical and histologic variants are known, including hypertrophic, atrophic, or pigmented AK. Although usually asymptomatic, some patients may experience local tenderness or a stinging sensation or may suffer from the cosmetic appearance of any lesions.

Correct diagnosis and therapy are crucial due to the possible progression of AK to cutaneous squamous cell carcinoma (cSCC) [1,2,3]. Annual rates of transformation vary from 0.1% to 25% [4], but spontaneous regression is also observed and can occur in 15–63% [3]. Currently, it is not possible to predict which AK transforms into cSCC due to a lack of valid biomarkers. Thus, early and consequent treatment is recommended in pertinent guidelines [5].

Solitary lesions are often treated with cryosurgery or curettage, whereas multiple lesions and surrounding field cancerization require field-directed treatment. This includes topical agents such as 5-fluorouracil, diclofenac in hyaluronic acid (HA), imiquimod, and photodynamic therapy [6]. In daily practice, a combination of treatment modalities is often used to maximize clearance rates, particularly in field disease and field cancerization [7].

In 2020, a formulation of tirbanibulin 1% ointment received FDA approval for the field treatment of AK [8], followed by approval by the EMA in 2021. Tirbanibulin acts as an inhibitor of tubulin polymerization and, as a secondary effect, Src kinase signalling, thereby inducing p53 expression, arrest of cell cycle progression, and mitotic activity in proliferating cells. Finally, it induces apoptosis but does not lead to a release of inflammatory cytokines, possibly explaining the good tolerability profile of tirbanibulin. It is applied once daily up to a 25 cm² area on the face or scalp for five consecutive days. In two pivotal phase III trials, complete patient and lesional clearance was reported in 44–54% and 76–82%, respectively [8]. A recent network meta-analysis of data from randomized controlled trials demonstrated that the efficacy and safety of tirbanibulin are in line with existing topical therapies for AK that have been approved in Europe [9]. However, data from a real-world setting outside of clinical trials are absent and the actinic keratosis area and severity index (AKASI) has not yet been applied to measure the efficacy of tirbanibulin.

In this study, we aimed to evaluate the outcome of tirbanibulin 1% ointment in the treatment of AK in a real-world setting. This is an important consideration as clinical trials may not always reflect the complexity and variability of patients seen in everyday practice.

## 2. Materials and Methods

In this single-centre study, adult patients with clinically typical, visible AK on the face or scalp were treated as per label with tirbanibulin 1% ointment. Eligibility criteria included age above 18 years and clinically typical AK lesions. To ensure no carry-over effects from previous treatments influenced our analysis, we strictly excluded patients who had undergone any of the following treatments in the 8 weeks preceding the start of tirbanibulin treatment: 5-fluorouracil 5% or 4% cream, imiquimod 5% or 3.75% cream, ingenol mebutate gel, diclofenac sodium/HA gel, photodynamic therapy, cryosurgery with liquid nitrogen, surgical excision, or chemical peelings.

Patients were provided with detailed instructions on the specific lesions to be treated with the tirbanibulin 1% ointment. Treatment was administered in accordance with the label once daily for 5 consecutive days on the same lesions or treatment field. The treatment outcome was evaluated after 4 weeks, patients were asked for adverse events after 1 week, and additional optional assessments were conducted at later time points. Due to the real-world setting, the duration of the second follow-up was not strictly defined and could vary among patients. A single investigator conducted lesion counts at baseline before treatment start and before follow-up visits. Patients were also instructed to report any local reactions and adverse events, along with the onset and resolution of such events.

Efficacy was measured using the AKASI score. The head was divided into four regions (scalp, forehead, left and right cheek with ear, chin and nose) and the percentage of the affected area in each region was assessed. Additionally, the severity was evaluated based on the three clinical signs, distribution, erythema, and thickness, resulting in a score from 0 to 18. AKASI scores were assessed pre-treatment and post-treatment. Patients were further assessed with digital dermoscopy using the FotoFinder^®^ imaging system (FotoFinder Systems GmbH, Bad Birnbach, Germany). In cases resistant to tirbanibulin treatment, a shave biopsy was performed for further histological examination and to rule out cSCC.

Local reactions were defined as erythema, flaking or scaling, crusting, swelling, vesiculation or pustulation, and erosions. The assessment of local reactions was carried out with the use of a semi-quantitative 4-point scale with scores of 0—absent, 1—mild, 2—moderate, and 3—severe.

Statistical analysis and graphical illustrations were calculated with GraphPad Prism^®^ 9 (GraphPad Software, Inc., San Diego, CA, USA). Paired t-tests were used to calculate *p*-values for pre- and post-treatment AKASI scores. Two-tailed *p*-values were calculated and considered significant with values *p* < 0.05. Violin plots were rendered for visual comparisons.

## 3. Results

### 3.1. Patient Population

Patients were included from November 2021 to February 2022. A total of 33 patients were found to be eligible for treatment and received a single course of tirbanibulin therapy. Three patients were lost to follow-up, and accordingly data from 30 patients were analysed (Table 1). 

All patients reported receiving all five daily doses of the treatment. After the first follow-up to assess outcomes at 4 weeks post-treatment, a second late follow-up was conducted in 23 patients after a mean of 3.7 months (range 1 to 6 months). Most patients were male (73%, *n* = 22) and had a skin type of I or II (90%, *n* = 27).

### 3.2. Efficacy

**Figure 1:**
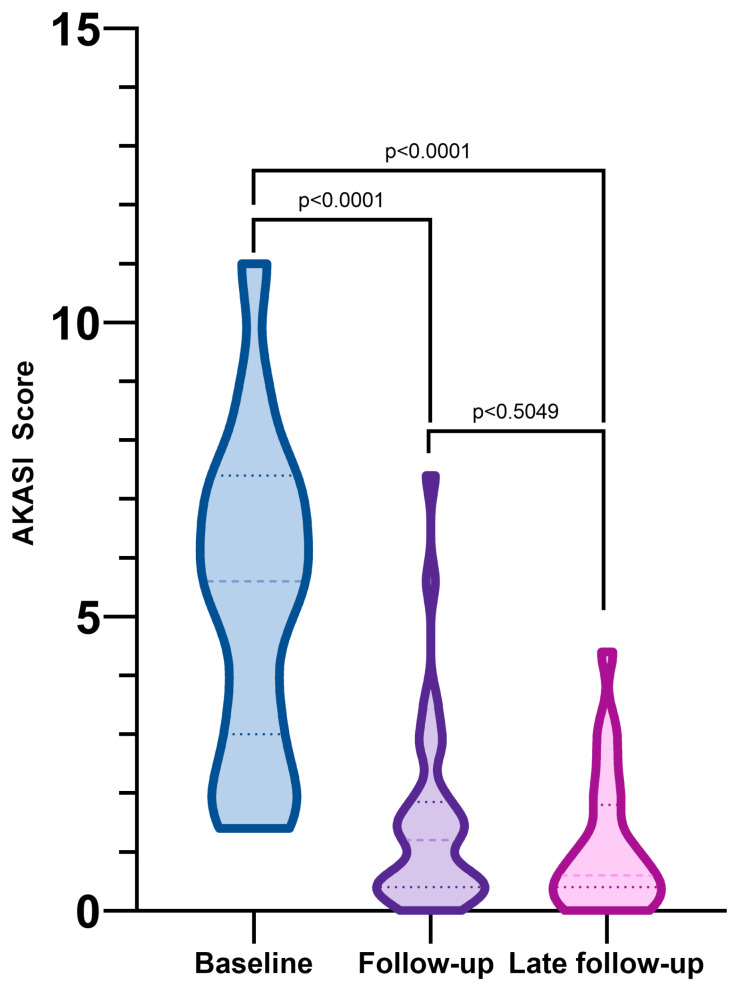
Comparison of median AKASI scores at baseline, 4-week follow-up, and last follow-up (mean 3.7 months) demonstrating significant reduction from a median score of 5.6 at baseline to 1.2 at 4-week follow-up (*p* < 0.0001).

In the 30 patients analysed, the median AKASI score was 5.6 (range 1.4–11) prior to treatment and 1.2 (range 0–7.4) post-treatment at the first follow-up (*p* < 0.0001; Figure 1).

**Figure 2:**
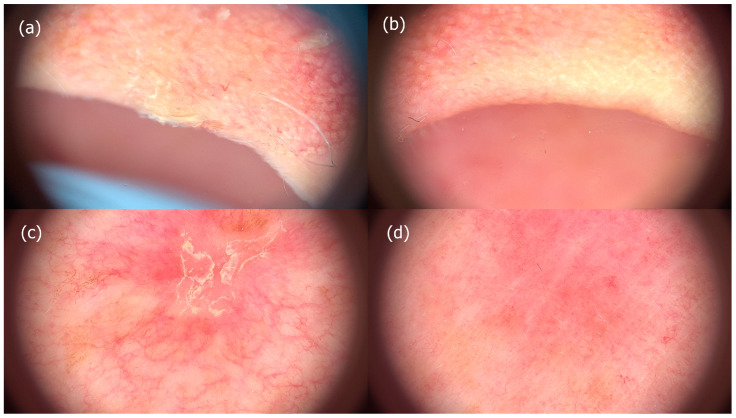
Comparison of clinical signs of actinic keratoses before and after treatment with tirbanibulin 1% ointment. Images (**a**,**c**) show the presence of scaliness and irritation at baseline, while images (**b**,**d**) demonstrate resolution of those signs post-treatment.

At the second follow-up, the median AKASI score was 0.6 (range 0–1.4). Complete clearance, as defined by AKASI scores less than 1, was achieved in 47% (*n* = 14) of patients at the first follow-up and in 57% (*n* = 13; 7 from a previous analysis lost to follow-up) at the second follow-up. In our study, no distinct differences were identified in the impact of tirbanibulin on distribution, erythema, or thickness. However, we observed that smaller lesions had a higher probability of healing with therapy. The highest post-treatment AKASI score was 7.4 in a patient with a pre-treatment AKASI score of 11. In addition to reducing AKASI scores, 97% of patients in this study showed a clinical response as seen by dermoscopy (Figure 2). 

**Figure 3:**
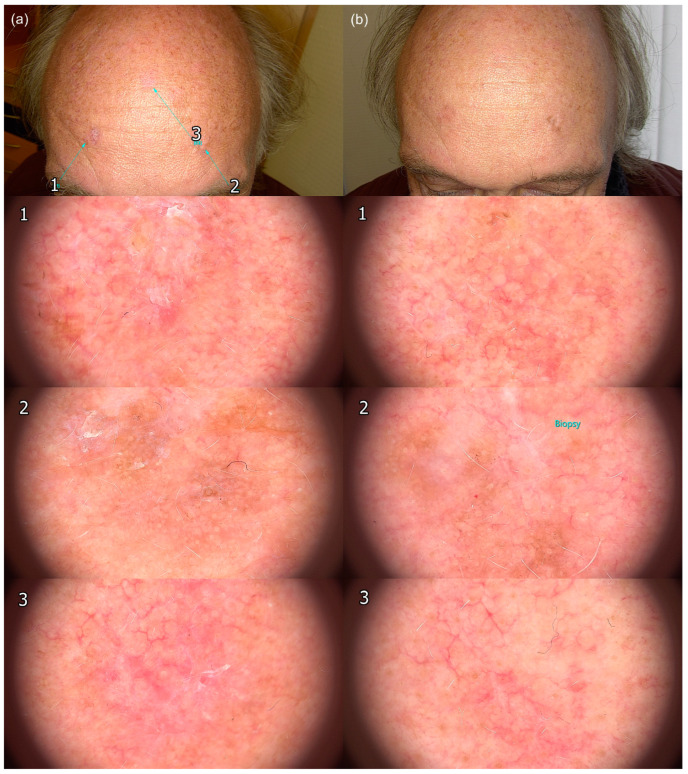
Clinical improvement of actinic keratoses with tirbanibulin 1% treatment. (**a**) Pre-treatment image showing multiple actinic keratoses on the face of the patient. (**b**) Post-treatment image taken 4 weeks later showing reduction in the appearance of actinic keratoses. However, a biopsy showed residual actinic keratoses on histological examination.

In cases with clinical evidence of residual lesions, a biopsy was performed (*n* = 10) (Figure 3). 

**Figure 4:**
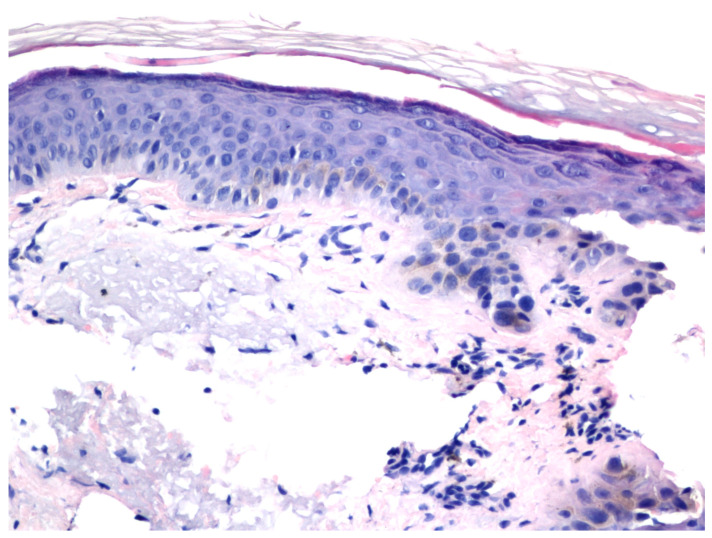
The microscopic examination of the biopsy taken from Figure 3, as seen in haematoxylin and eosin staining, revealed enlarged cells and atypical and hyperchromatic nuclei, indicative of residual actinic keratosis. (H&E: ×200).

One patient underwent a biopsy of a treatment-resistant lesion. Histopathologic examination revealed residual AK (Figure 3 and Figure 4). 

**Figure 5:**
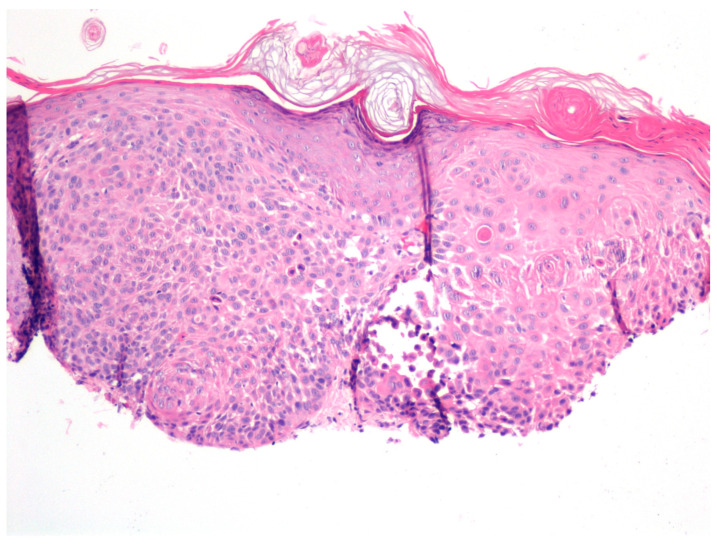
Microscopic examination of a biopsy from a therapy-resistant lesion revealed early invasive squamous cell carcinoma of the skin. (H&E: ×100).

In another patient with a treatment-resistant lesion, the biopsy revealed early invasive cSCC (Figure 5).

### 3.3. Local Adverse Events

**Figure 6:**
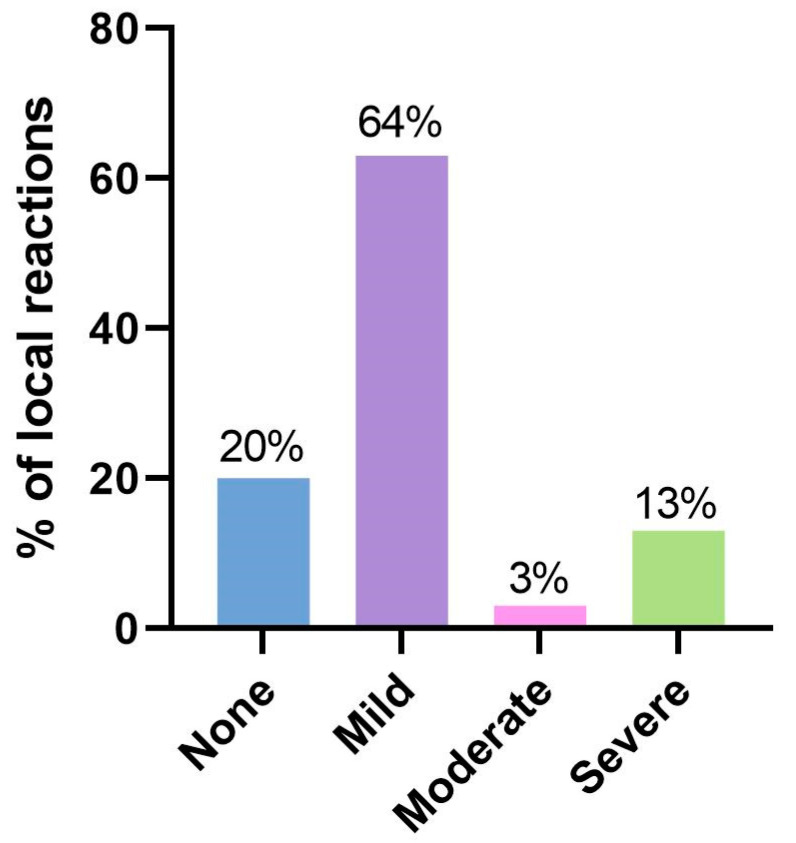
Distribution and severity of local reactions in patients undergoing treatment.

Local reactions associated with the treatment were reported to occur between 2 and 10 days after the initiation of therapy. One patient reported mild pruritus on the second day of treatment. The median onset of adverse events was on the seventh day, and the mean time for resolution was 5 days. All local reactions resolved spontaneously and without sequelae. The most common local reactions were erythema (80%, *n* = 26) and flaking or scaling (43%, *n* = 13). Less frequently observed local reactions were pustulation (7%, *n* = 2) and pruritus (7%, *n* = 2). No local reactions were observed in six patients (20%; Figure 6). Our analysis did not identify any correlation between the reduction in AKASI and the occurrence of local adverse reactions, suggesting that the degree of inflammation did not necessarily predict a better response to treatment.

## 4. Discussion

In this study, the outcome of tirbanibulin 1% ointment was evaluated as a treatment for AK in a real-world setting. Tirbanibulin was approved for the treatment of AK based on data from two pivotal phase III trials performed in the USA [8]. Tirbanibulin was approved by the EMA in July 2021. However, real-world evidence for the outcomes of tirbanibulin treatment in AK has not yet been reported to the best of our knowledge. In this study, 30 patients were evaluable after one treatment cycle with tirbanibulin. The efficacy was measured with the AKASI, which integrates distinct facets such as anatomical region, size of the affected area, and clinical signs of AK and represents an easy-to-use quantitative measurement tool. The AKASI is more accurate than the sole counting of lesions and was previously used to assess the treatment effects of topical diclofenac sodium in HA and photodynamic therapy [10,11,12]. However, the effects of tirbanibulin on the AKASI scores of treated patients have not yet been investigated. Results from this study showed that most patients had a significant reduction in AKASI scores at two follow-up time points. In the pivotal studies, efficacy was assessed at day 57 after treatment, which was approximately 4 weeks after the first evaluation in this study. The AKASI reduction was clearly and significantly observed as soon as 4 weeks after treatment, implying rapid lesion clearance after treatment initiation. We defined complete clearance as an AKASI score of less than 1. In contrast, the definition of complete clearance used in the pivotal studies by Blauvelt et al. [8] was a 100% reduction in the number of visible lesions in the treatment field, which was assessed 57 days after treatment. Despite this difference in definition and timing, the rates of complete clearance were comparable between our study (47%) and the two trials reported by Blauvelt et al. (44% and 54%). This clearance rate is in line with existing topical treatments for AK available in Europe in indirect comparisons [9]. At the second follow-up after a median of 3.7 months, complete clearance was even slightly higher, suggesting that the treatment of tirbanibulin are not short-lived as was implied by the rather high recurrence rates observed in the pivotal trials.

In terms of safety, we did not report any new adverse events in our study. The most common local reaction in our study was erythema (80%), which is similar to the rate reported by Blauvelt et al. [8] (91%). The second most common local reaction in our study was flaking or scaling (43%), which was less frequent than in the trials reported by Blauvelt et al. [8] (82%). All local reactions resolved spontaneously and without sequelae, demonstrating the good tolerability of tirbanibulin, which is in line with data from other trials [13].

Currently, there are no head-to-head trials comparing tirbanibulin to other commonly used topical treatments for AK, such as fluorouracil, diclofenac/HA, imiquimod, and destructive therapies. Therefore, it is difficult to compare the efficacy of tirbanibulin to these other agents. Furthermore, the long-term data on the efficacy of tirbanibulin are still lacking, as the current studies only followed patients for up to 1 year [8]. Real-world evidence is urgently needed to further investigate tirbanibulin as a novel topical drug and to define its role in the competitive treatment landscape of AK. Large-scale non-interventional studies are currently underway and actively recruiting.

We acknowledge certain limitations in our study that should be considered while interpreting the results, such as the relatively short follow-up period, the small sample size, and the potential for selection bias due to patients’ previous therapies.

In conclusion, tirbanibulin 1% ointment shows promise as a treatment option for AK with high clearance rates and low incidence of severe local reactions. The results from this study from a European population are highly consistent with the data from the pivotal trials. However, further studies with longer follow-up periods and direct comparisons to other commonly used topical agents are needed to fully evaluate the long-term efficacy and cost-effectiveness of tirbanibulin for the treatment of AK.

## Figures and Tables

**Table 1:** Patient characteristics of the study cohort (*n* = 30).

Median age (years)	74 (64–92)
Gender	
Female	8 (27%)
Male	22 (73%)
Skin type	
I	10 (33%)
II	17 (57%)
III	2 (7%)
IV	1 (3%)
Prior therapies	
Curettage/cryosurgery	8 (27%)
Chemical peeling	10 (33%)
Diclofenac in hyaluronic acid	12 (40%)
5-Fluorouracil	8 (27%)
Imiquimod	1 (3%)
Laser-assisted photodynamic therapy	1 (3%)
Ingenol mebutate	2 (7%)
Unknown therapy or none	12 (40%)

## Data Availability

Data are unavailable due to privacy.

## References

[1] Fernandez Figueras M.T. (2017). From Actinic Keratosis to Squamous Cell Carcinoma: Pathophysiology Revisited. J. Eur. Acad. Dermatol. Venereol..

[2] Werner R.N., Stockfleth E., Connolly S.M., Correia O., Erdmann R., Foley P., Gupta A.K., Jacobs A., Kerl H., Lim H.W. (2015). Evidence- and Consensus-Based (S3) Guidelines for the Treatment of Actinic Keratosis—International League of Dermatological Societies in Cooperation with the European Dermatology Forum—Short Version. J. Eur. Acad. Dermatol. Venereol..

[3] Werner R.N., Sammain A., Erdmann R., Hartmann V., Stockfleth E., Nast A. (2013). The Natural History of Actinic Keratosis: A Systematic Review. Br. J. Dermatol..

[4] Marks R., Rennie G., Selwood T.S. (1988). Malignant Transformation of Solar Keratoses to Squamous Cell Carcinoma. Lancet.

[5] Leiter U., Heppt M.V., Steeb T., Amaral T., Bauer A., Becker J.C., Breitbart E., Breuninger H., Diepgen T., Dirschka T. (2020). S3 Guideline for Actinic Keratosis and Cutaneous Squamous Cell Carcinoma (CSCC)—Short Version, Part 2: Epidemiology, Surgical and Systemic Treatment of CSCC, Follow-up, Prevention and Occupational Disease. J. Dtsch. Dermatol. Ges..

[6] Goldenberg G. (2017). Treatment Considerations in Actinic Keratosis. J. Eur. Acad. Dermatol. Venereol..

[7] Steeb T., Wessely A., Leiter U., French L.E., Berking C., Heppt M.V. (2020). The More the Better? An Appraisal of Combination Therapies for Actinic Keratosis. J. Eur. Acad. Dermatol. Venereol..

[8] Blauvelt A., Kempers S., Lain E., Schlesinger T., Tyring S., Forman S., Ablon G., Martin G., Wang H., Cutler D.L. (2021). Phase 3 Trials of Tirbanibulin Ointment for Actinic Keratosis. N. Engl. J. Med..

[9] Heppt M.V., Dykukha I., Graziadio S., Salido-Vallejo R., Chapman-Rounds M., Edwards M. (2022). Comparative Efficacy and Safety of Tirbanibulin for Actinic Keratosis of the Face and Scalp in Europe: A Systematic Review and Network Meta-Analysis of Randomized Controlled Trials. J. Clin. Med..

[10] Schmitz L., von Dobbeler C., Gupta G., Gambichler T., Szeimies R.M., Morton C.A., Dirschka T. (2018). Photodynamic Therapy Leads to Significant Improvement of Actinic Keratosis Area and Severity Index (AKASI). Photodiagnosis Photodyn. Ther..

[11] Pellacani G., Gupta G., Micali G., Malvehy J., Stratigos A.J., Casari A., Chester J., Kaleci S., Dirschka T. (2018). Actinic Keratosis Area Severity Index (AKASI): Reproducibility Study and Comparison with Total Lesion Count. Br. J. Dermatol..

[12] Schmitz L., Gupta G., Segert M.H., Kost R., Sternberg J., Gambichler T., Stockfleth E., Dirschka T. (2018). Diclofenac Sodium 3% in Hyaluronic Acid 2.5% Gel Significantly Diminishes the Actinic Keratosis Area and Severity Index. Ski. Pharmacol. Physiol..

[13] Dosik J., Cutler D.L., Fang J., Padullés L. (2022). Contact Sensitization and Phototoxic and Photoallergic Potential of Tirbanibulin 1% Ointment in Healthy Volunteers. JID Innov. Ski. Sci. Mol. Popul. Health.

